# Supplementary material for: Unravelling mutational signatures with plasma circulating tumour DNA
Source: Nat Commun. 2024 Nov 14;15:9876. doi: 10.1038/s41467-024-54193-2 (PMC11564803; doi:10.1038/s41467-024-54193-2)
Supplement: Supplementary file 1 — Supplementary Information [file 41467_2024_54193_MOESM1_ESM.pdf]

Unravelling mutational signatures with plasma circulating tumour DNA

Supplementary Figures

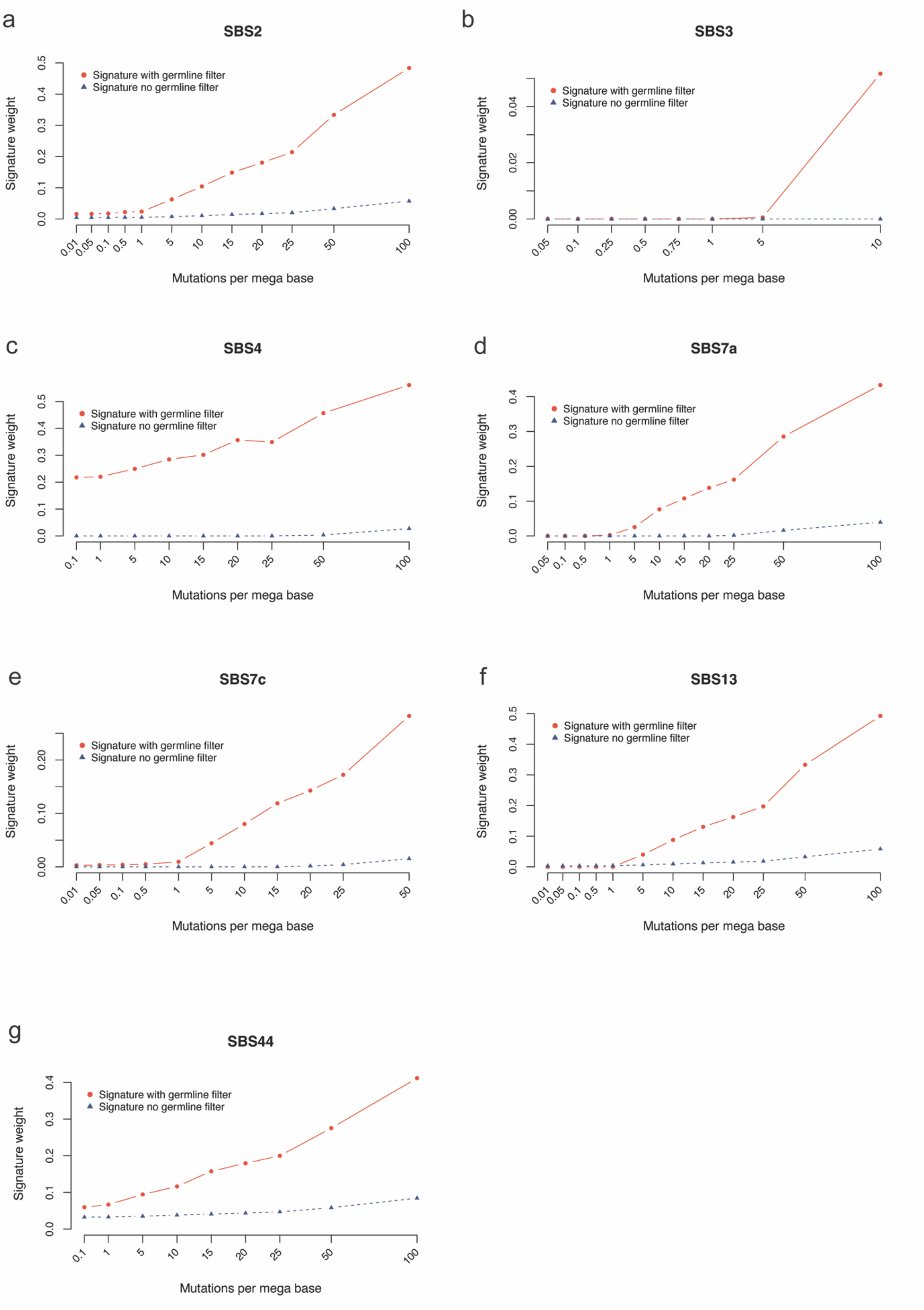

Supplementary Fig. 1, Effect of gnomAD germline variant filtering on signature detection. Assessed for the **(a)** APOBEC signature SBS2, **(b)** HRD signature SBS3, **(c)** smoking signature SBS4, **(d)** UV damage signatures SBS7a **(e)** SBS7c, **(f)** APOBEC signature SBS13 and **(g)** dMMR signature SBS44 in LCWGS data simulated with varying mutational burdens based on the COSMIC database, with depth of coverage fixed at 3x. Source data are provided as a Source Data file.

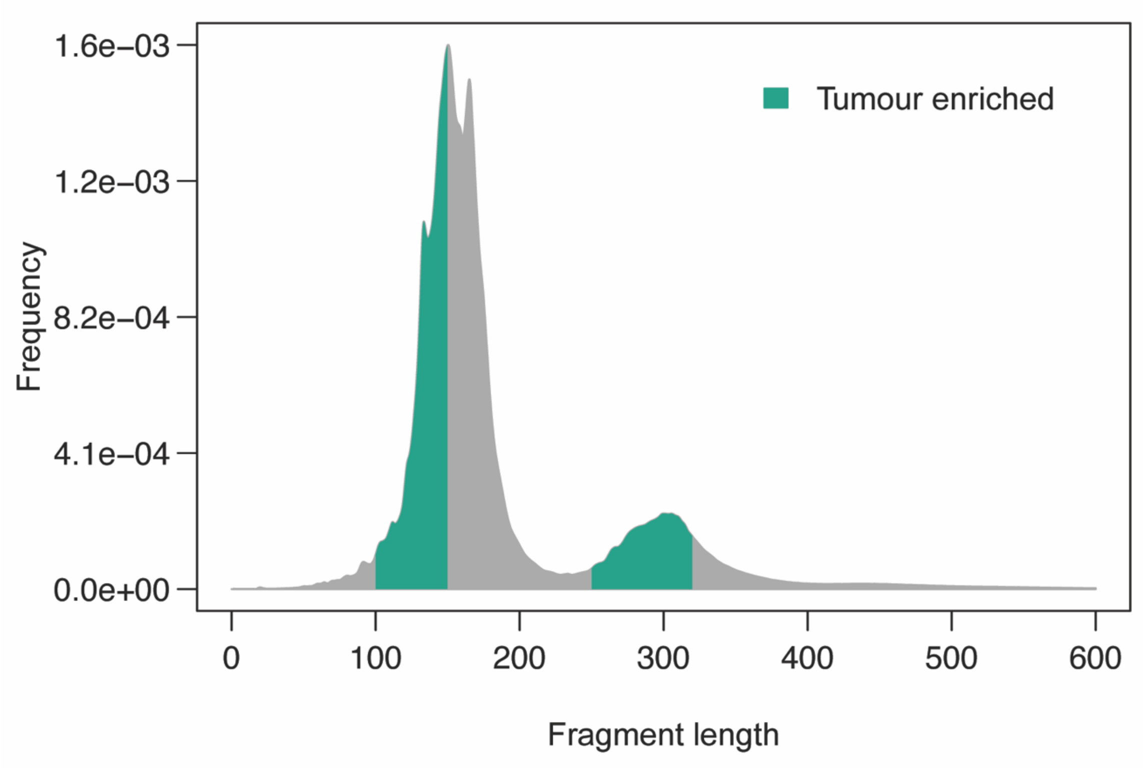

Supplementary Fig. 2 A characteristic circulating DNA fragment length profile from plasma LCWGS. The highlighted size ranges (100-150 bp, 250-325 bp) are typically enriched for ctDNA.

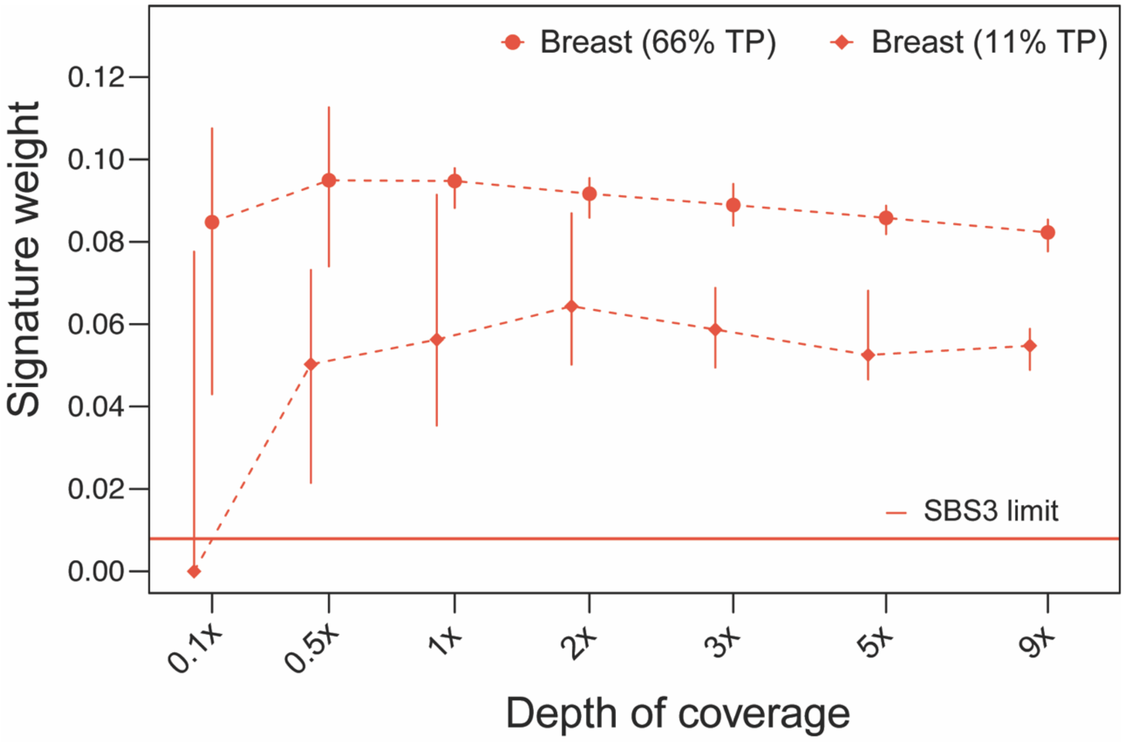

Supplementary Fig. 3 Effect of sequencing coverage on the limit of SBS3 signature detection in two *BRCA*-mutant breast cancer patients with estimated tumour purities of 66% and 11% in plasma. Each vertical line represents a boxplot of the signature weights of 20 *in-silico* replicates. The bounds of each vertical line are the 25th to 75th percentile and the median signature weights are denoted by the symbols. The horizontal line denotes the detection threshold for SBS3 derived from 60 healthy plasma controls. Source data are provided as a Source Data file.

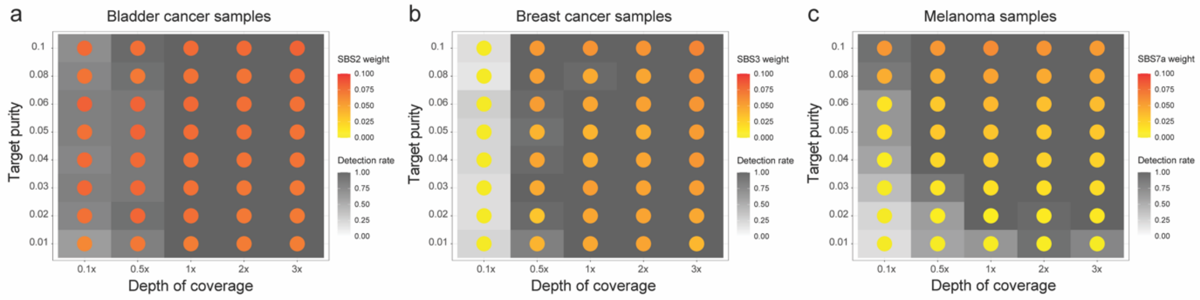

Supplementary Fig. 4 The combined effect of sequencing coverage and tumour purity on the limit of signature detection was assessed for signatures SBS2, SBS3 and SBS7a from **(a)** a bladder cancer, **(b)** a *BRCA*-mutant breast cancer and **(c)** a melanoma patient respectively. The x-axis denotes different depths of coverage, and the y-axis denotes different target purities. Each cell summarises across 20 *in-silico* ctDNA-healthy admixtures of different combinations of depth of coverage and target tumour purities. Median signature weights of the 20 replicates are denoted by the colour of the circles. The level of grey denotes the proportion in each 20 *in-silico* replicate grouping where the signature was detected. Detection thresholds per signature type and coverage/purity combination were derived from 60 healthy plasma controls. Source data are provided as a Source Data file.

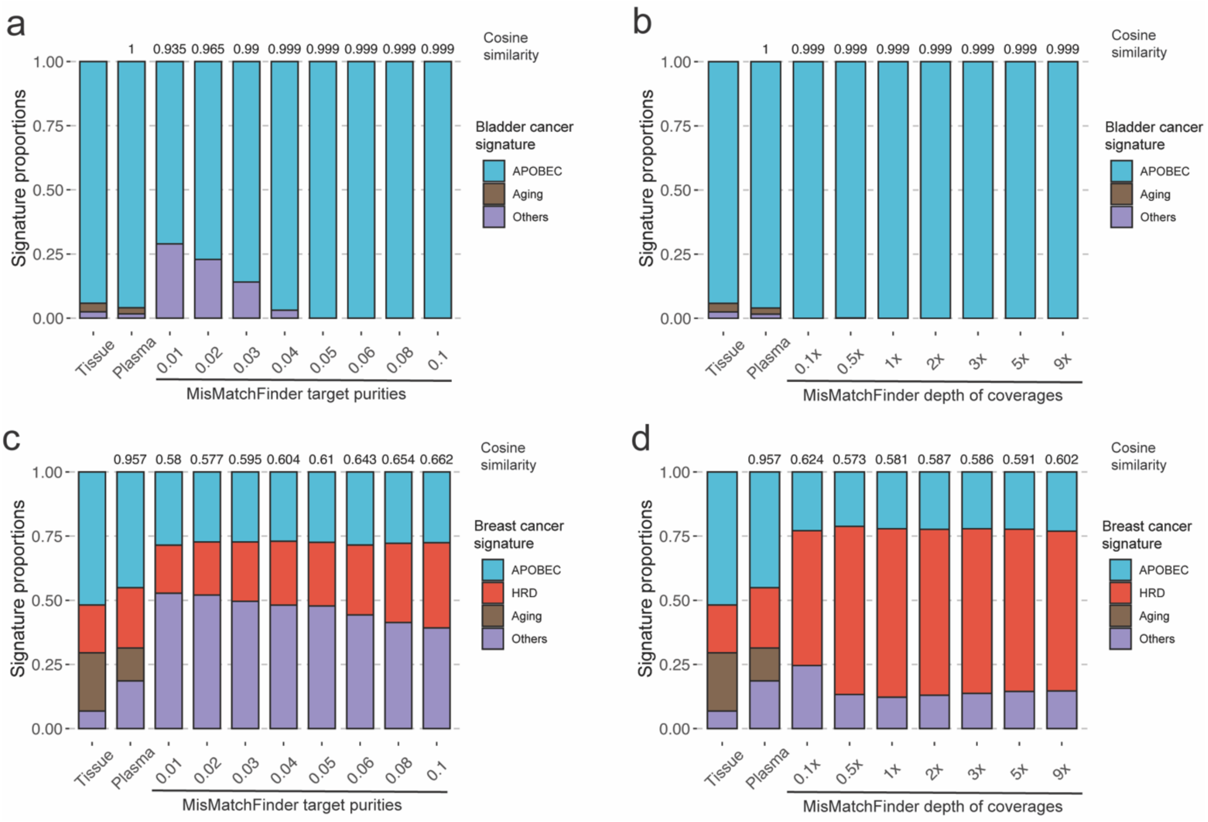

Supplementary Fig. 5 Comparison of grouped SBS mutational signatures detected from paired tumour tissue and plasma in **(a,b)** a bladder cancer patient and **(c,d)** a *BRCA1*-mutant breast cancer patient across varying tumour purities and sequencing depth. The first two columns of signatures were obtained from somatic variants called using 1) high-coverage paired tumour tissue-germline data and 2) plasma-germline data. The rest of the columns are signature analysis results from variants inferred from low-coverage plasma data without a germline control using MisMatchFinder. The signatures assessed were those which have been previously found in bladder cancers (APOBEC: SBS2, SBS13; Aging: SBS1, SBS5; Others: SBS8, SBS29, SBS40) and breast cancers[1] (APOBEC: SBS2, SBS13; HRD: SBS3; Aging: SBS1, SBS5; Others: SBS8, SBS9, SBS17a, SBS17b, SBS18, SBS37, SBS40, SBS41). Source data are provided as a Source Data file.

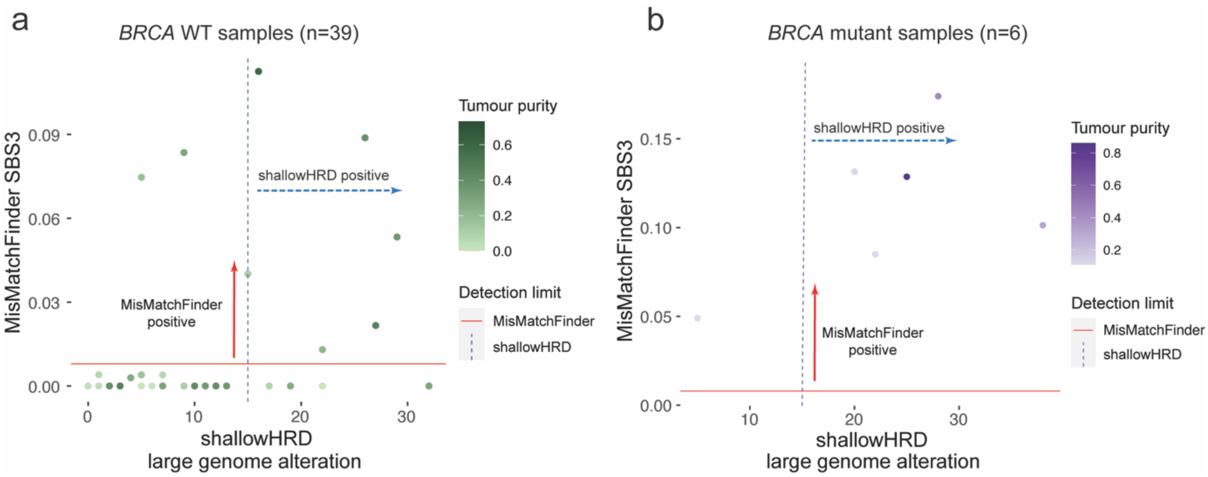

Supplementary Fig. 6. Comparison of MisMatchFinder and shallowHRD for HRD signature detection in 45 breast cancer patients. HRD detection in **(a)** 39 patients with no mutations detected in *BRCA1/2* genes (*BRCA* WT) and **(b)** 6 patients with *BRCA1/2* mutations (*BRCA* mutant). Each dot represents one patient coloured by its estimated tumour purity in plasma. The detection thresholds of MisMatchFinder and shallowHRD are marked as a horizontal solid red line and vertical dashed blue line respectively. Source data are provided as a Source Data file.

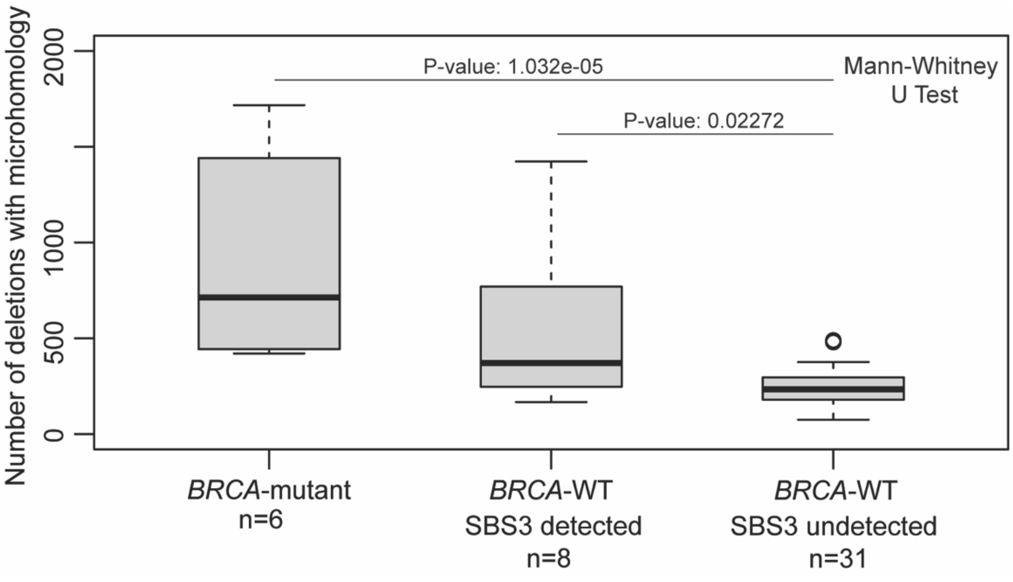

Supplementary Fig. 7. The number of deletions with microhomology for 45 breast cancer patients with known *BRCA1/2* mutational status and SBS3 detection status using MisMatchFinder. Box plots indicate median (middle line), 25th to 75th percentile (box) and 1.5 times the inter-quartile range from the first and third quartiles (whiskers). Dots denote sample outliers. Source data are provided as a Source Data file.
