# Supplementary material for: Unravelling mutational signatures with plasma circulating tumour DNA
Source: Nat Commun. 2024 Nov 14;15:9876. doi: 10.1038/s41467-024-54193-2 (PMC11564803; doi:10.1038/s41467-024-54193-2)
Supplement: Supplementary file 3 — Description of Additional Supplementary Files [file 41467_2024_54193_MOESM3_ESM.pdf]

Description of Additional Supplementary Files

Supplementary Data 1. Meta data and signature detection for 45 breast cancer plasma samples and 40 melanoma plasma samples. BRCA-WT: No mutations found in BRCA1/2 genes; BRCA-mutant: germline mutations detected in BRCA1/2 genes. "0" and "1" indicates if signature has not been detected (0) or detected (1) by MisMatchFinder. All signatures related to sequencing artefacts have been removed.

Supplementary Data 2. Meta data and signature detection for 16 colorectal cancer plasma samples and 21 healthy plasma samples. MSI_status denotes microsatellite instability status in the colorectal cancer patients. "0" and "1" indicates if signature has not been detected (0) or detected (1) by MisMatchFinder. All signatures related to sequencing artefacts have been removed.

Supplementary Data 3. Meta data and signature weights for 271 cancer samples and 262 healthy controls in the pan-cancer plasma low-coverage, whole genome sequencing cohort. Signature weights were re-normalised after removing sequencing artefact related signatures.
