# Supplementary material for: Unravelling mutational signatures with plasma circulating tumour DNA
Source: Nat Commun. 2024 Nov 14;15:9876. doi: 10.1038/s41467-024-54193-2 (PMC11564803; doi:10.1038/s41467-024-54193-2)
Supplement: Supplementary file 5 — Reporting Summary [file 41467_2024_54193_MOESM5_ESM.pdf]

nature portfolio

Corresponding author(s): Sarah-Jane Dawson
Dineika Chandrananda

Last updated by author(s): Oct 29, 2024

Reporting Summary

Nature Portfolio wishes to improve the reproducibility of the work that we publish. This form provides structure for consistency and transparency in reporting. For further information on Nature Portfolio policies, see our Editorial Policies and the Editorial Policy Checklist.

Statistics

For all statistical analyses, confirm that the following items are present in the figure legend, table legend, main text, or Methods section.

n/a	Confirmed	
☐	☒	The exact sample size (*n*) for each experimental group/condition, given as a discrete number and unit of measurement
☐	☒	A statement on whether measurements were taken from distinct samples or whether the same sample was measured repeatedly
☐	☒	The statistical test(s) used AND whether they are one- or two-sided<br>*Only common tests should be described solely by name; describe more complex techniques in the Methods section.*
☒	☐	A description of all covariates tested
☒	☐	A description of any assumptions or corrections, such as tests of normality and adjustment for multiple comparisons
☐	☒	A full description of the statistical parameters including central tendency (e.g. means) or other basic estimates (e.g. regression coefficient) AND variation (e.g. standard deviation) or associated estimates of uncertainty (e.g. confidence intervals)
☐	☒	For null hypothesis testing, the test statistic (e.g. *F*, *t*, *r*) with confidence intervals, effect sizes, degrees of freedom and *P* value noted<br>*Give P values as exact values whenever suitable.*
☒	☐	For Bayesian analysis, information on the choice of priors and Markov chain Monte Carlo settings
☒	☐	For hierarchical and complex designs, identification of the appropriate level for tests and full reporting of outcomes
☒	☐	Estimates of effect sizes (e.g. Cohen's *d*, Pearson's *r*), indicating how they were calculated

Our web collection on statistics for biologists contains articles on many of the points above.

Software and code

Policy information about availability of computer code

Data collection: No software was used for data collection

Data analysis: A custom software tool (MisMatchFinder, version 0.1.0) was developed within this study to analyse mutational signatures from low coverage sequencing data from plasma circulating DNA. We offer MisMatchFinder as a fully compiled static binary file as well as the source codes and libraries used within the tool in the following repository:
https://atlassian.petermac.org.au/bitbucket/projects/SJDAW/repos/mismatchfinder/browse
The sequencing reads of samples used in our study were mapped to human genome GRCh38 by bwa-mem (version 0.7.17) with alternate-contig aware mapping. Presumed PCR and optical duplicates were marked using the Picard tools software suite (version 2.17.3).
The mutational signature fitting analysis in this study was done using sigminer (v2.3.0). To establish the detection limits of each signature from a panel of healthy controls, beta distributions were fitted with R package fitdistrplus (v1.1-8). To perform gold-standard tumour-germline paired variant calling, the DRAGEN pipeline (v4.2.4) was applied in this study. VarSCAT (v1.1.0) was used to label variants as "deletions in microhomology". ShallowHRD (v1.13) was used in this study to compare the performance with MisMatchFinder, of which the input was generated by QDNAseq (v1.32.0).
The data analysis in our study was done with R (4.2.0). The cosine similarity was measured with R package lsa (version 0.73.3).

For manuscripts utilizing custom algorithms or software that are central to the research but not yet described in published literature, software must be made available to editors and reviewers. We strongly encourage code deposition in a community repository (e.g. GitHub). See the Nature Portfolio guidelines for submitting code & software for further information.

Data

Policy information about availability of data

All manuscripts must include a data availability statement. This statement should provide the following information, where applicable:

- Accession codes, unique identifiers, or web links for publicly available datasets
- A description of any restrictions on data availability
- For clinical datasets or third party data, please ensure that the statement adheres to our policy

The pan-cancer LCWGS dataset used in this study is available from the European Genome-phenome Archive (EGA) under accession number: EGAD00001005339 [https://ega-archive.org/datasets/EGAD00001005339]. The signature weights for this cohort are contained within Supplementary Data 3. The sequencing data for the 16 colorectal cancer patients with known microsatellite instability status as well as 21 healthy individuals is available from EGA under accession number EGAS00001006377 [https://ega-archive.org/studies/EGAS00001006377]. The signature detection information for this cohort is contained within Supplementary Data 2. The sequencing data from breast cancer and melanoma patients as well as the in-house healthy controls are available under EGA accession numbers EGAS00001007593 [https://ega-archive.org/studies/EGAS00001007593] and EGAS50000000569 [https://ega-archive.org/studies/EGAS50000000569]. The signature detection information for these cancer samples is contained within Supplementary Data 1. The tissue, germline and plasma sequencing for the bladder cancer case is available from EGA under accession number EGAS50000000452 [https://ega-archive.org/studies/EGAS50000000452]. Access will be granted by application to the relevant Data Access Committees for each cohort, and will be governed by the provisions laid out in the associated informed consent for each cohort and the terms contained in the relevant Data Access Agreements. Other data required for filters such as gnomAD variants for the germline filter, high mappability regions as white-listed regions used in this study can be found in the following Zenodo repository: https://doi.org/10.5281/zenodo.13845728. Source data are provided with this paper.

Research involving human participants, their data, or biological material

Policy information about studies with human participants or human data. See also policy information about sex, gender (identity/presentation), and sexual orientation and race, ethnicity and racism.

Reporting on sex and gender	Sex and gender were not considered in the study design as this information was not necessary for the particulars of the study.
Reporting on race, ethnicity, or other socially relevant groupings	N/A
Population characteristics	N/A
Recruitment	45 patients with breast cancer, 40 melanoma patients and 60 healthy controls were recruited following informed consent with each study approved by the Peter MacCallum Cancer Centre Human Research Ethics Committee (Breast HREC 15/72; Melanoma HREC 11/105 and 07/38; Healthy controls HREC 98/36 and 17/56).
Ethics oversight	Peter MacCallum Cancer Centre Human Research Ethics Committee

Note that full information on the approval of the study protocol must also be provided in the manuscript.

Field-specific reporting

Please select the one below that is the best fit for your research. If you are not sure, read the appropriate sections before making your selection.

☒ Life sciences ☐ Behavioural & social sciences ☐ Ecological, evolutionary & environmental sciences

For a reference copy of the document with all sections, see nature.com/documents/nr-reporting-summary-flat.pdf

Life sciences study design

All studies must disclose on these points even when the disclosure is negative.

Sample size	The clinical cohorts utilized were bio specimen collections not clinical trial data and this study can be considered as a retrospective analysis. Hence, there was no sample size calculation carried out for this study.
Data exclusions	No patients or samples were specifically excluded in this study.
Replication	The evaluation of our method was done with 20 in-silico replications for each biological sample.
Randomization	No randomization was performed as this was a retrospective analysis of existing data.
Blinding	No blinding was performed as this was a retrospective analysis of existing data.

Reporting for specific materials, systems and methods

We require information from authors about some types of materials, experimental systems and methods used in many studies. Here, indicate whether each material, system or method listed is relevant to your study. If you are not sure if a list item applies to your research, read the appropriate section before selecting a response.

Materials & experimental systems

n/a	Involved in the study
☒	☐ Antibodies
☒	☐ Eukaryotic cell lines
☒	☐ Palaeontology and archaeology
☒	☐ Animals and other organisms
☒	☐ Clinical data
☒	☐ Dual use research of concern
☒	☐ Plants

Methods

n/a	Involved in the study
☒	☐ ChIP-seq
☒	☐ Flow cytometry
☒	☐ MRI-based neuroimaging

Plants

Seed stocks	N/A
Novel plant genotypes	N/A
Authentication	N/A
